# Surgery

**Published:** 1893-04

**Authors:** John Parmenter


### SURGERY.

By JOHN PARMENTER, M. D.

### UNJUST CONDEMNATION OF CATGUT.

Dr. Lanphear (*International Journal of Surgery*, January, 1893,) in an article upon the Unjust Condemnation of Catgut, after discussing some recent papers upon the unreliability (from the aseptic standpoint) of catgut, proceeds to give his own experiences with this material. To this end he has summarized the results of fifty-four operations of various kinds, in only three of which did suppuration occur. He concludes his paper as follows: "I am sure that any surgeon will be convinced of the superiority of catgut to all other suture materials in proper place, if perfect antiseptic precautions be taken and good gut be used. Carbolized catgut is totally unreliable. I prepare my own catgut thus: Good, strong, gut, free from flaws, is washed in soap and water until all the "kink" is out; it is then dried with a towel, wound loosely around the fingers and dropped into a jar of sulphuric ether, C. P., in which it lies for forty-eight hours; it is then transferred to a jar containing oil of juniper, in which it floats from two days to a week, according to size; it is then wound—with fingers surgically clean—on spools and kept in alcohol ninety per cent. and oil of juniper ten per cent., carefully sealed. This is perfectly safe."

[In our experience, no mixture for the final preservation of catgut equals oil of juniper three parts, and alcohol one part, with the addition of hydro-naphthol five grains to the ounce of the mixture. The gut thus preserved is very flexible, does not become weakened in strength, and has a pleasant odor.—P.]

---

### VACCINATION.

Cathell (*Maryland Medical Journal,* January 21, 1893,) suggests the following to prevent the very bad arm that often follows vaccination with bovine virus. Those in charge of the patient should try daily to raise the scab after the twenty-first day and remove it as soon as it is found to be detachable; next to mop and dry the sore, letting the air glaze the exposed surface before re-applying the clothes. When the edges are loose, but the crust is held by a strong and fleshy core at the center, the loosened margin should be cut away with scissors, including as much as possible of the central core itself. The sore is then dressed with a salve or dusted with aristol, iodoform, or europhen, if necessary. The writer thus concludes: "This not only prevents the excessively sore arm and chronic ulcer, with its burrowing, corroding, and tissue-absorbing pus, with the huge and unsightly keloidal cicatrix, but also secures the characteristic scar with its series of minute depressions or pits which unmistakably prove that it is the relic of a genuine Jennerian vesicle."

---

### WOUNDS OF THE HEART.

Foy (*Lancet,* * * * ,) makes the following interesting observations upon wounds of the heart, a subject full of medico-legal importance: In considering the subject, I think the natural division of such wounds is—(1) Wounds of the ventricles, and (2) wounds of the auricles, these being subdivided into (*a*)

penetrating and (*b*) non-penetrating. Such an arrangement seems to have been followed by the older writers, commencing with M. Morand, whose work, Observation sur une Plaie du Cœur, was published in 1735. Garé noticed (Opera Omnia, liber viii.,) that wounds of the ventricle cause death slowly. The great significance of this is dealt with and clearly shown by Beck in his Jurisprudence. Baron Dupuytren gives Latour's case of "a ball extracted from the right ventricle near to the apex of the heart, and partly covered by the pericardium," six years after the injury was inflicted. William Harvey found a bullet in the heart of a stag. Even wounds that penetrate the auricle are not necessarily fatal. Dr. Grace, of Fife's case, lasted two days after puncture of the right auricle, and Surgeon Billy's case—wound of the right ventricle—survived five days, the longest period on record that I know of. Death may, however, very suddenly result from the wound, for, although the Duc de Berry survived his injury some hours, King Henry IV. died almost immediately from the stab of the assassin Ravaillac. A very slight puncture of the auricle may quickly cause death. Two such instances are on record: (1) The Sardinian nobleman's case, in 1728, at the Court of Victor Amadeus; and (2) that of Admiral Villeneuve, who on April 22, 1807, killed himself by piercing the right auricle of his heart with a fine needle. But, perhaps, the most remarkable case on record is that reported in the *Manitoba Lancet*, Vol. I., and copied by me in my article, The Literature of Punctured and Lacerated Wounds of the Heart, in which an attempt was made to remove by operation a needle from the interior of the right ventricle of a medical student. The operation was unsuccessful, but the manipulation of the heart displaced the needle from the position in which it was causing great distress, and for years the patient suffered no inconvenience."

---

### HYDROGEN PEROXIDE IN THE TREATMENT OF CARBUNCLE.

GOLDEN (*Medical Record*, February 25, 1893,) reports a case in which hydrogen dioxide was employed in the treatment of carbuncle. Parenchymatous injections (with an ordinary hypodermic syringe) of the peroxide (M. xxv., xxx.,) were made twice a day. In from six to eight hours new openings formed, giving exit to considerable quantities of liquified core and relieving tension. The writer thinks the new drain channels due to the evolution of gas (due to oxidation within the core) which was forced through the points of least resistance. It is, in the writer's opinion, superior to carbolic acid and "a very useful agent in the treatment of this troublesome affection."

